# Structural health monitoring of aircraft through prediction of delamination using machine learning

**DOI:** 10.7717/peerj-cs.1955

**Published:** 2024-03-27

**Authors:** Rajeswari D, Osamah Ibrahim Khalaf, Srinivasan R, Pushpalatha M, Habib Hamam

**Affiliations:** 1 Department of Data Science and Business Systems, School of Computing, College of Engineering and Technology, SRM Institute of Science and Technology, Kattankulathur, Tamilnadu, India; 2 Department of Solar, Al-Nahrain Research Center for Renewable Energy, Al-Nahrain University, Jadriya, Baghdad, Iraq; 3 Department of Computing Technologies, School of Computing, College of Engineering and Technology, SRM Institute of Science and Technology, Kattankulathur, Tamil Nadu, India; 4 Université de Moncton, Moncton, Canada

**Keywords:** Structural health monitoring, Delamination, Prediction, Stack ensemble, Machine learning

## Abstract

**Background:**

Structural health monitoring (SHM) is a regular procedure of monitoring and recognizing changes in the material and geometric qualities of aircraft structures, bridges, buildings, and so on. The structural health of an airplane is more important in aerospace manufacturing and design. Inadequate structural health monitoring causes catastrophic breakdowns, and the resulting damage is costly. There is a need for an automated SHM technique that monitors and reports structural health effectively. The dataset utilized in our suggested study achieved a 0.95 R2 score earlier.

**Methods:**

The suggested work employs support vector machine (SVM) + extra tree + gradient boost + AdaBoost + decision tree approaches in an effort to improve performance in the delamination prediction process in aircraft construction.

**Results:**

The stacking ensemble method outperformed all the technique with 0.975 R2 and 0.023 RMSE for old coupon and 0.928 R2 and 0.053 RMSE for new coupon. It shown the increase in R2 and decrease in root mean square error (RMSE).

## Introduction

The structure of the aircraft is made up of composite materials because of its well-known properties like excellent resistance to fatigue, high strength, weight, high modulus, and stiffness. The carbon composite materials are widely used for manufacturing the aircraft structure (Yue & Aliabadi, 2020). However, the composite materials in the structure are damaged due to aging, fatigue, dynamic load, and cyclic load. Structural health monitoring (SHM) plays a vital role in identifying these damages. Inadequate SHM leads to catastrophic failures and the damages caused by catastrophic failure is costly (Xu et al., 2017). The factors to be considered for SHM are strain pattern, fiber failure, matrix cracking, delamination, and skin stiffener (Larrosa, Lonkar & Chang, 2014). This work concentrates on delamination.

The lamination is a collection of laminae. In the structure of aircraft, the lamination has 14 laminas each has 1 to 1.1 mm thickness. Laminae is a positioning of unidirectional or woven fibers in a matrix. The fibers are act as a load carrying agent and commonly strong and stiff. The purpose of the matrix is to protect the fibers by distributing the load across it. The layers of amination are made by same matrix material. Due to some interlaminar stresses available on the structure, the lamination starts to delaminate. Gradually it spreads to entire structure. The extant of delamination worsen the characteristics of composite material and finally leads to the failure in aircraft structure (Seno & Aliabadi, 2022).

To overcome this failure, the structural health of aircraft should be monitored on timely basis. The current SHM techniques are complicated and time consuming as it is a manual process. This manual SHM requires more resources, *i.e*., human resources, time and cost. Also, the disassembling and assembling of aircraft structure increases downtime. There is a need for an automated SHM techniques that monitors and reports structural health efficiently. This work focuses on automated SHM techniques and optimizes design, increases safety, reduces downtime, maintenance time and cost.

This work monitors the guided lamb waves in piezoelectric sensor network to identify the delamination in the aircraft structure. Sensor signal features like frequency, load, cycle, time of flight (ToF) and power spectral density (PSD) are used to quantify the damage. machine learning (ML) algorithm is one of the best techniques to predict the delamination size (Liu et al., 2021). In this work, ML based prediction approaches are used to calculate the delamination size. Stack ensemble with linear regression as a meta model and nearest neighbour, extra tree, AdaBoost, gradient boost and decision tree algorithm is implemented. The work contributes the following things:

1. The sensor features like PSD, ToF and interrogation frequency are calculated for various composite coupons from given actuator and sensor signal.
2. The delamination size was calculated from given X-ray images of multiple composite coupons and considered as ground truth.
3. An ensemble regression technique is used with five base level models to predict the size of delamination.

## Literature review

Toyama et al. (2005) presented the variation in stiffness of carbon fiber reinforced polymers (CFRP) laminates using guided lamb waves. The quantitative damage of laminates was calculated by *in situ* quantification of the wave velocity. It provides more location accuracy than other conventional technique. Johnson & Chang (2001) introduced the two-part verification to find the stiffness and strength of composite laminates. The first part represents the characterization of matrix crack which helps for damage progression. The second part calculated the amount of damage. The proposed technique has been implemented using computer code, PDcell. Saxena et al. (2011) experimented how the delamination are influencing on the velocity of guided lamb wave. The density of matrix crack in a particular path and delamination was identified using local regression technique in Su, Ye & Lu (2006). Larrosa, Lonkar & Chang (2014) and Larrosa et al. (2011) classified and predicted damage. These researchers clearly indicate the effective use of ML algorithms to classify the data generated from the piezoelectric actuators in the surface of composite materials. Nevertheless, there is no clear method to calculate the delamination size, which is the objective of this work.

ML and deep learning techniques are also used for infrastructure health monitoring. Lei et al. (2017) proposed multi-task architecture and ensembleDetNet technique to detect and classify infrastructure damage. This technique improved 5% accuracy than other state-of-art detection and classification technique. Liu et al. (2017) represented faster R-CNN technique with RestNet101 architecture to detect and measure external damage in historic masonry buildings. This proposed methodology identified spalling and efflorescence damage with 0.950 and 0.999 respectively. ASTM International (2000a) used ultrasonic becons instead of GPS in unmanned aerial vehicles (UAV) and CNN for damage identification. This method processed video data collected by UAV and produced 91.9% sensitivity and 97.7% specificity.

Currently ML algorithms are used to analyse the relationship among the features available in a data set is used to predict the damage (ASTM International, 2000b). The delamination prediction problem is formulated as regression problem. However, much work not been carried out on expansion of delamination using ML method. To address this problem Liu et al. (2017) experimented to find the length of the path around the delamination instead of calculating the delamination area (Huston, 1994) by ML methods. This technique provided the solution to the overfitting problem in modelling phase. Though it provides the results in acceptable range, exact calculation of delamination size is remained unsolved. However, the prediction rate of delamination needs to be improved. This work focuses on this.

**Figure 1:**
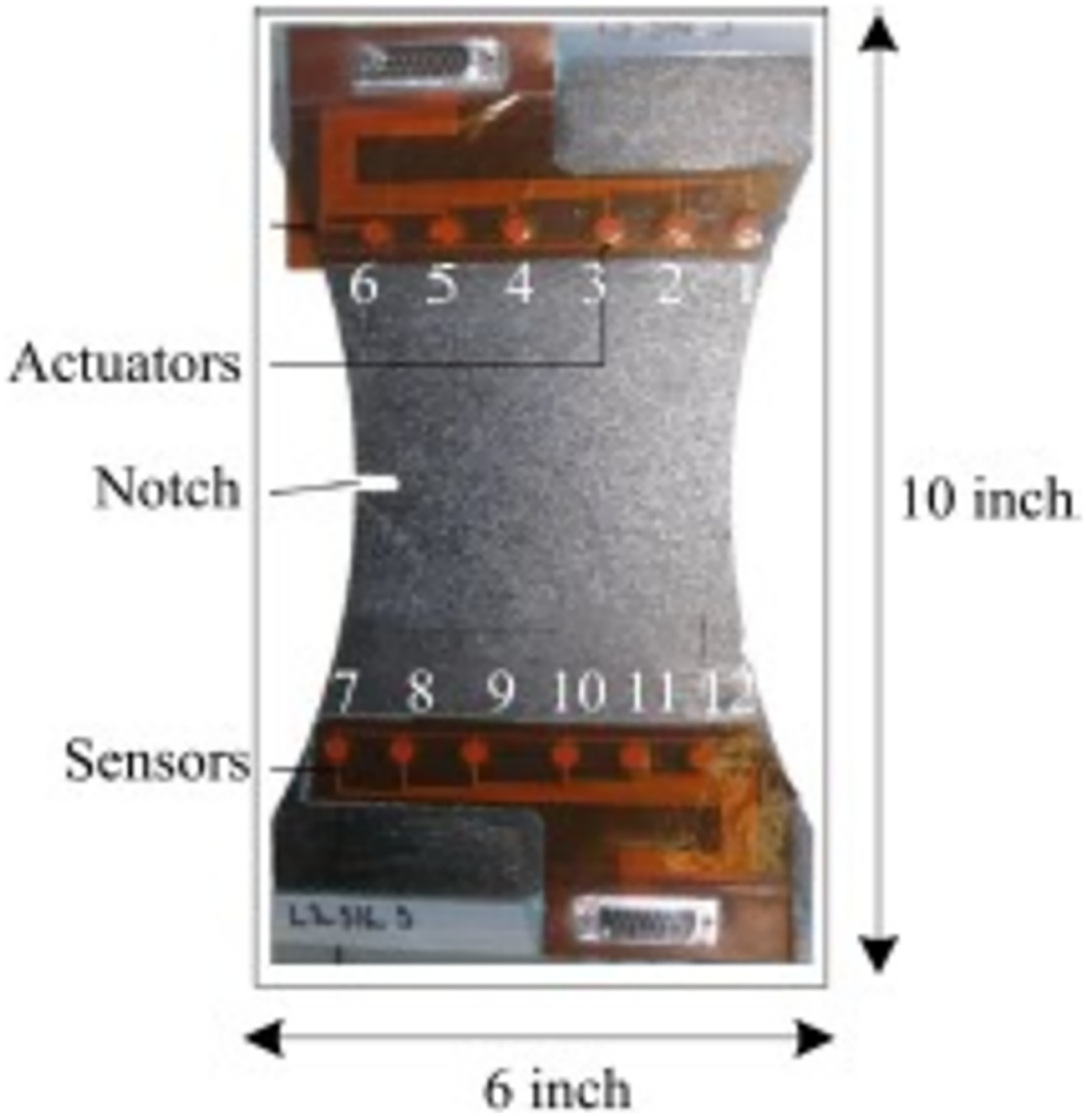
Composite coupon.

NASA performed experiments of fatigue aging on CFRP using following ASTM standards D3039 (Rohit, Chandrawat & Rajeswari, 2021) and D3497 (Sikka et al., 2022). The test was done by using Torayca T700G. These materials are used in aircraft and sports goods which needs high property of composite materials. In composite materials, weight of the surface is 600 g/m^2^, fabric thickness is 0.90 mm, density is 1.80 g/cm^3^ and tensile strength is 4.900 MPa and it is called as coupon. Finally, it is fabricated and divided into 10-inch length and 6-inch-wide piece is presented in Fig. 1 (Liu et al., 2021).

Huston narrated the effects of fatigue in unidirectional carbon fiber reinforced proxy using residual stiffness and strength model (Ma et al., 2017). These results are compared with Chiachio et al. (2013) result. The authors taken the fatigue cycling test for each 50,000 cycles then collect the Piezoelectric Transducer (PZT) sensor data of 36 trajectories and seven interrogation frequencies. The outcome of the fatigue test is (1) malfunction data collection for actuator-to-sensor system (2) delamination size quantification (3) analyse the variation among coupons. All these outcomes are considered into account for this work. The sample coupon in Fig. 1 has six actuators and six sensors. The lamb waves are disseminated from actuator and sensed by sensors. To calculate the delamination area X-ray images are used and to initiate the delamination at a point, notch with necking geometry was used.

To the best of my knowledge, existing research uses ML techniques to forecast delamination in aircraft structures. The proposed method predicts the size of the delamination using the ensemble algorithm, which combines one or more ML approaches. Furthermore, the computation of delamination size from X-ray is automated.

## Materials and Methods

### Dataset description

**Figure 2:**
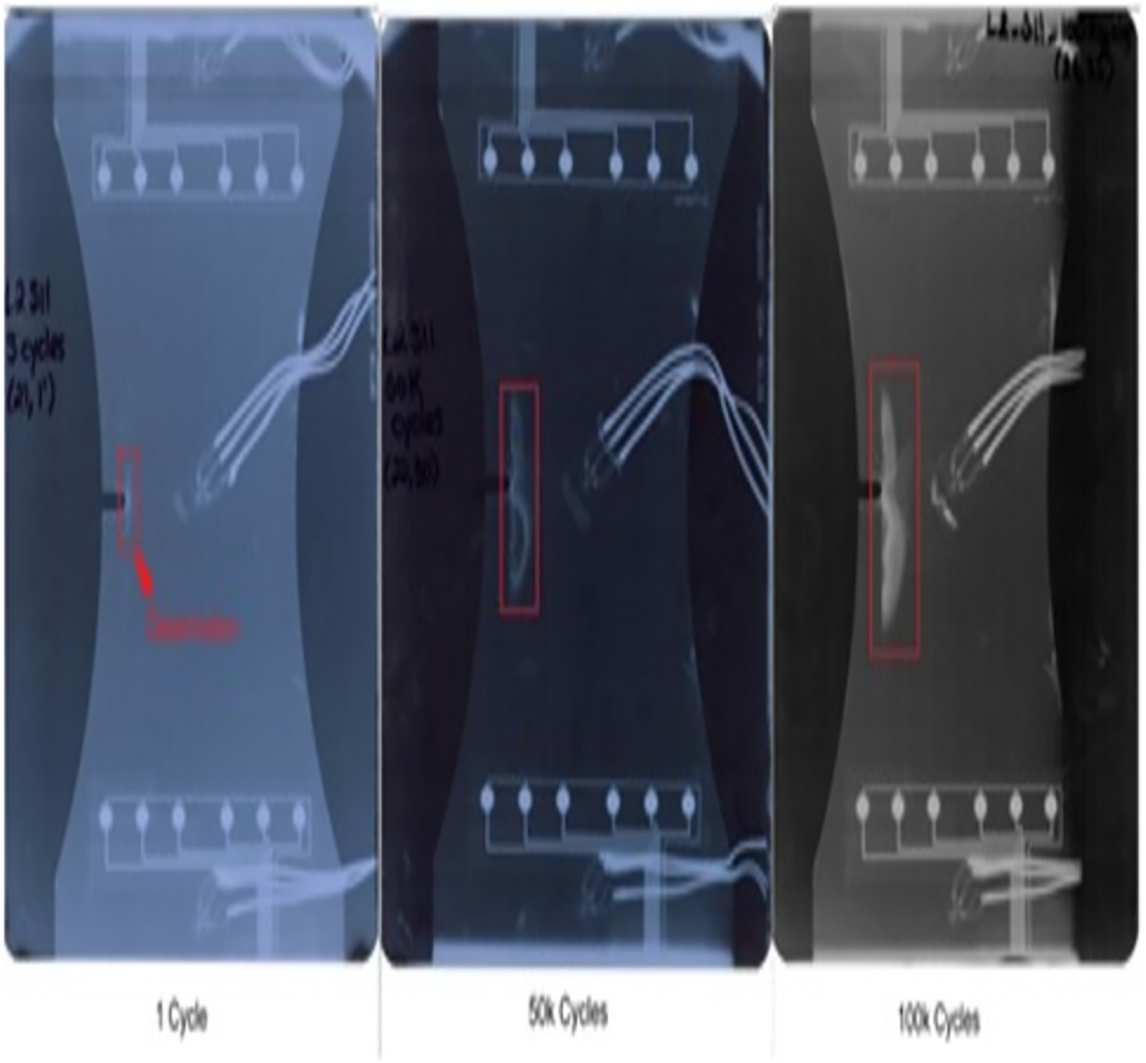
X-ray images for various loading cycles.

The dataset used in this article is downloaded from NASA Ames Research Center. It is a CFRP materials dataset. It clearly indicates that size of delamination is direct proportional to loading cycle, which was calculated against fatigue cycling. To improve the efficiency of experimental data the calculation was repeated number of times. Figure 2 represents the X-ray image of composite coupon at 150,000 and 100,000 loading cycles, sequentially.

**Figure 3:**
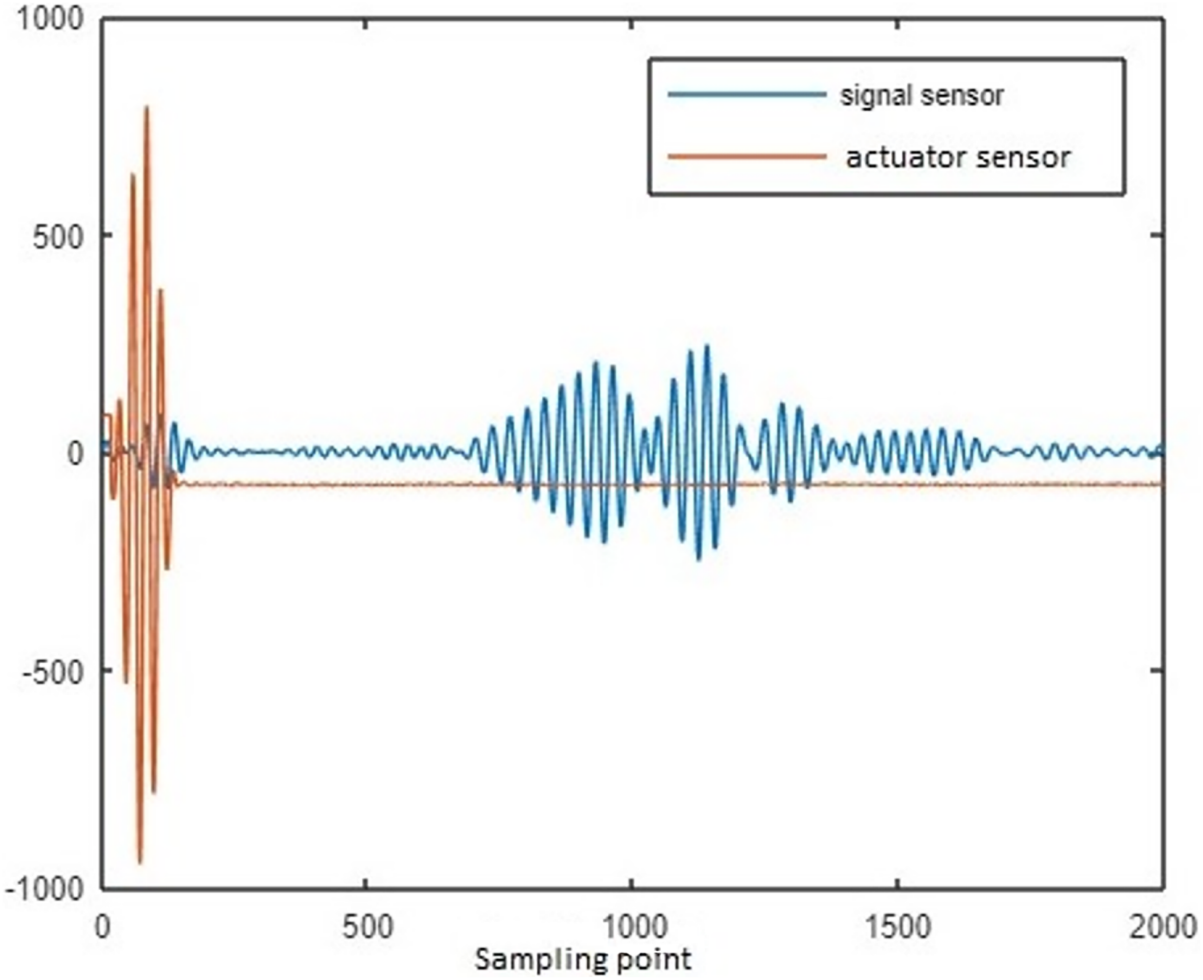
Sensor and actuator signal for delamination data of CFRP coupon.

The lamb waves are disseminated along the coupon surface to identify the delamination interrogation. The waves propagated through the delamination area has change in its strength while reaching the sensor. The delamination size is increased for increase in loading cycle. While the delamination is increase, the signal strength reaches through the delamination path is reduced. The changes in spectral amplitude of time and frequency domain intimates the delamination on the surface of coupon. To calculate the delamination size, sensor signal features loading cycle, interrogation frequency, power spectral density (PSD) and time of flight (ToF) are considered in this article. To characterize the property of materials the above features are mostly used by the researchers (Larrosa, Lonkar & Chang, 2014; Toyama et al., 2005). Figure 3 represents the raw sensor and actuator signal for data of CFRP coupon.

Loading cycle: To get the sensor signal for various load, fatigue test is done on composite coupon. The output of every 10,000 cycle is recorded. The actuator and sensor signal for various loading cycle is given in the NASA dataset.

Interrogation frequency: To decompose the actuator and sensor signal into various frequency spectrums, Fast Fourier Transform (FFT) is used. The input frequency correlate with high amplitude is considered as interrogation frequency.

Power spectral density: PSD for various frequencies is calculated using FFT by the function of time. The peak in the PSD values is reduced by increase in delamination size. However, the strength of the signal input is reduced due to wave scattering in delamination area.

Time of flight: The time difference between the actuator signal peak and sensor signal peak is ToF.

**Figure 4:**
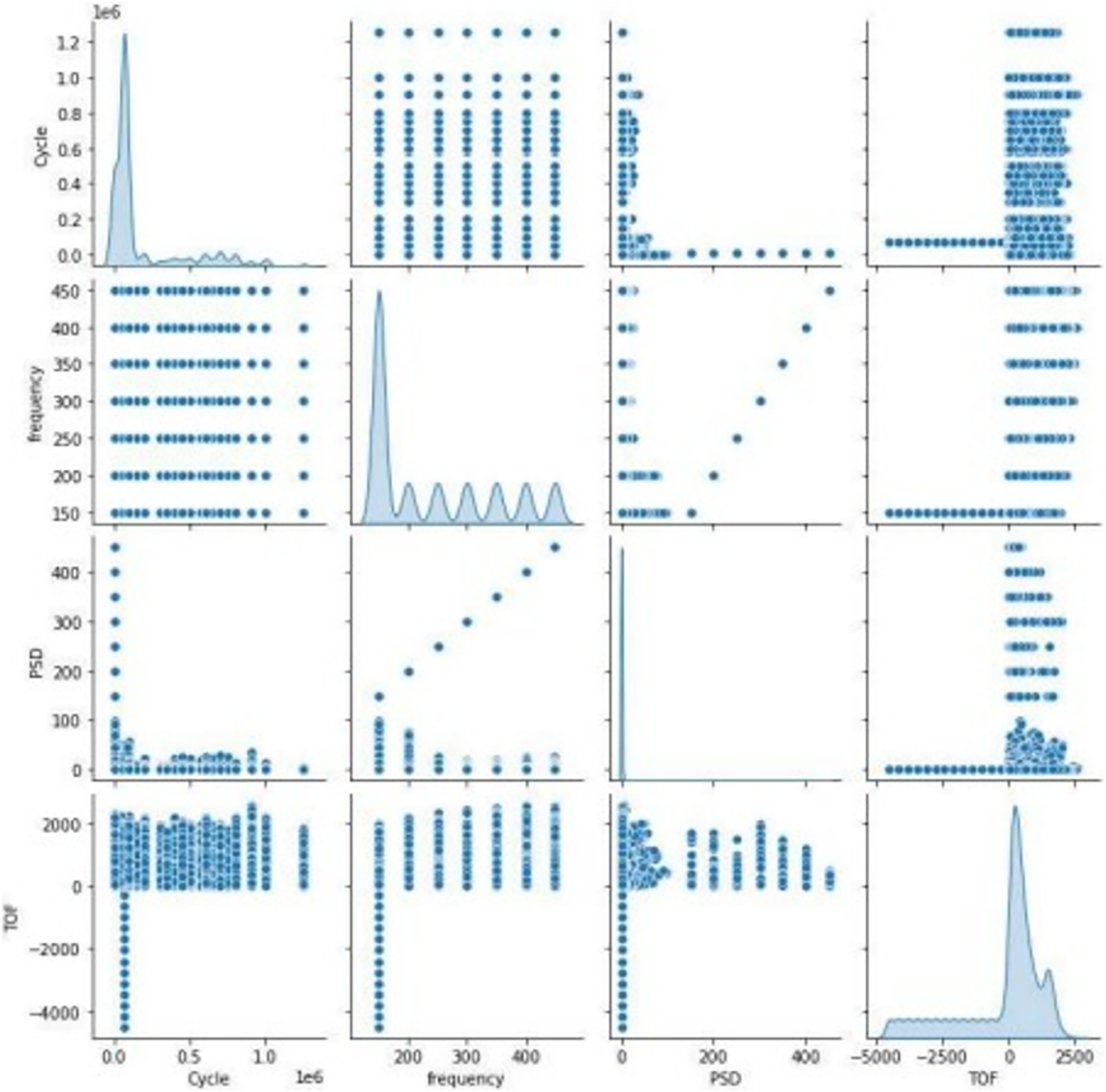
Correlation between features.

Figure 4 represents the association between features in the dataset with scatter plots depends on the correlation matrix method is shown in Eq. (1).

(1) $${X_{mn}} = \; \displaystyle{{\mathop \sum \nolimits_{i = 1}^p \left( {{m_i} - \; \bar m} \right)\left( {{n_i} - \; \bar n} \right)} \over {\sqrt {\mathop \sum \nolimits_{i = 1}^p {{\left( {{m_i} - \; \bar m} \right)}^{2\; }}\mathop \sum \nolimits_{i = 1}^p {{\left( {{n_i} - \; \bar n} \right)}^2}} }}$$

The x_mn_ is correlation coefficient, m and n are random variables and $\bar m$ and $\bar n$ are the means of m and n. The scattering of sensor signal feature is replicated on left axis and bottom axis and the diagonal represents the density plot of the feature.

**Figure 5:**
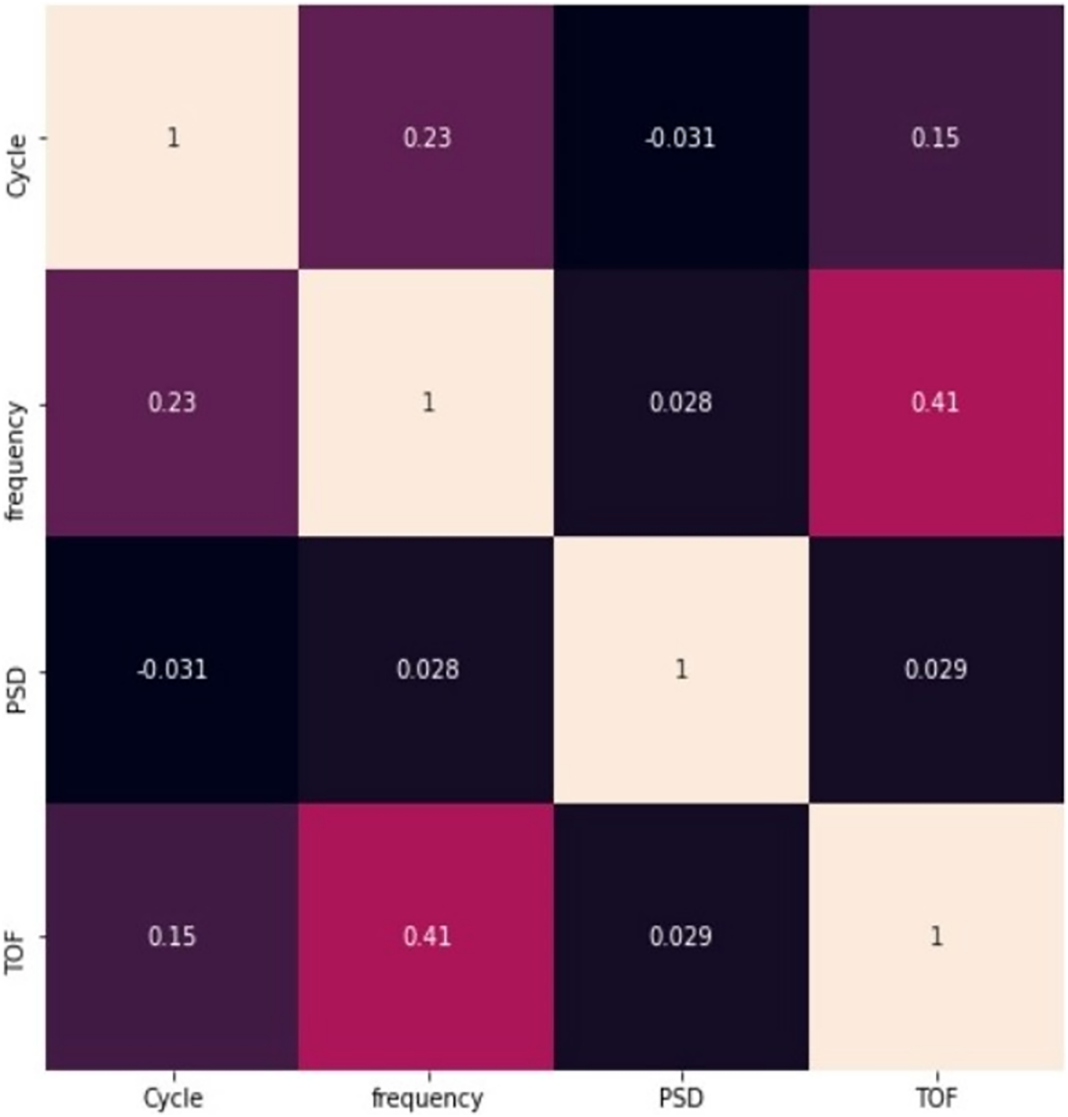
Relationship between pair of features.

Figure 5 represents the correlation between the pair of features. None of the correlation value exceeds 0.8, it clearly indicates that the features are not closely correlated with each other, and all the features are taken into account for further process. The figure also represents that there is a negative correlation between cycle and PSD.

MATLAB is used to process the raw data given by NASA to obtain the specified features. To calculate the ground truth (*i.e*., delamination size) Area property of region props method is used on X-ray images with delamination in MATLAB. Finally, the dataset has 150,949 data points with six features like cycle, load, frequency, PSD, ToF and ground truth.

### Delamination size prediction using machine learning

**Figure 6:**
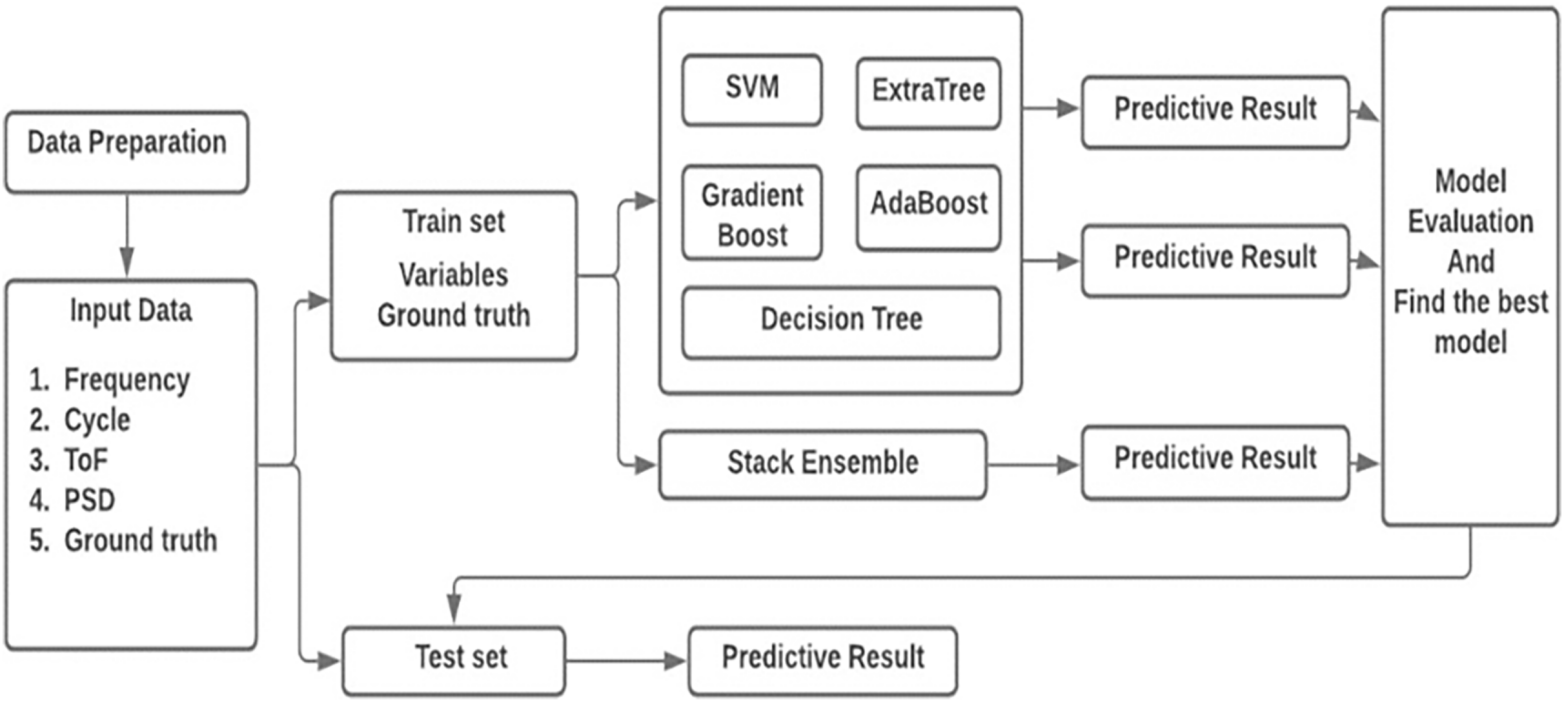
Architecture of the proposed model.

In this work, sensor signal features acquired from composite coupon is used to predict the delamination size. A deterministic technique is entrenched by regression investigation which permits the diagnostic values obtained by independent variable n specified the dependent variables m_x_. Figure 6 shows the workflow of the prediction technique. The four sensor features are formed as the vector m_x_ = [m_1_, m_2_, m_3_, m_4_] = [cycle, frequency, PSD, ToF] as input to the prediction method. Delamination size is used as the ground truth n.

In recent years, ML algorithms are widely used to predict the delamination size regression problem and provided the best results (Liu et al., 2021). Consequently, this work implemented the regression models like support vector machine (SVM), extra tree, gradient boost, AdaBoost and decision tree and finally, stack ensemble technique is used to improve the prediction accuracy.

**Support vector machine**: The SVM is basic and widely used prediction technique. Due to SVM’s scalable capability, it can be well suited to small datasets. With the help of loss function SVM can be applied to prediction problems. In this work, SVR (support vector regression) with ‘rbf’ kernel is used. The degree of polynomial kernel method is set as 3, kernel coefficient for ‘rbf’ is set as scale, value for gamma is set as 1/(n_features * X.var()) and stopping condition tolerance is set as 1e-3 by default. Kernal size used for this implementation is set as 200 MB. SVM regression technique is presented in Algorithm 1 (Rätsch, Onoda & Müller, 2001).

**Algorithm 1:** SVM model.

**Result:** Prediction of delamination size
**Input:** Sensor features with ground truth $\left( {{m_p},{n_p}} \right)_{p = 1}^x$
**1** clf = svr (m_p_, n_p_)
2 clf.fit (k = 'rbf', degree = 3, g = 'scale', tol = 0.001, C = 1.0, c_size = 200, m_it = −1)
**Output:** SVM Prediction model

In the above algorithm k represents kernel, g represents gamma, c_size represents cache_size and m_it represents maximum iteration.

**Extra tree model:** The extra tree model contains number of prediction trees capitulated from various training data (Soni, Arora & Rajeswari, 2020). Every tree is considered as self-prediction method and average of every prediction tree’s output gives the final regression. Extra tree regression technique is presented in Algorithm 2. The increase in number of prediction trees yields to better performance. In this work, the amount of prediction tree available in forest is 100, mean squared error criterion, the amount of samples needed in leaf node is 1, amount of samples needed to divide in internal node is 2 are used.

**Algorithm 2:** Extra tree model.

**Result:** Prediction of delamination size
**Input:** Sensor features with ground truth $\left( {{m_p},{n_p}} \right)_{p = 1}^x$
**1** clf = ExtraTreeRegressor (m_p_, n_p_)
2 clf.fit (n_est = 100, c = 'squared_error', m_s_s = 2, m_s_l = 1, max_features = 'auto')
**Output:** Extra Tree Prediction model

In the above algorithm n_est represents n_estimators, c represents criterion, m_s_s represents min_sample_split, m_s_l represents min_samples_leaf and m_ft represents max_features.

**Gradient boosting model**: Gradient boosting is a supplement model in an onward step-wise technique. It permits for improvement of random differentiable loss method. At every epoch a prediction tree is fit on the negative gradient of the specified loss method (Rajeswari et al., 2022). Gradient boosting technique generate a regression technique in the structure of an ensemble of weak regression technique. Gradient boosting regression technique is presented in Algorithm 3. Squared error loss function is used for regression. The contribution of prediction tree shrinks by learning rate and it was set as 0.1. The increase in boosting epoch provides good performance and it was set as 100.

**Algorithm 3:** Gradient boosting model.

**Result:** Prediction of delamination size
**Input:** Sensor features with ground truth $\left( {{m_p},{n_p}} \right)_{p = 1}^x$
**1** clf = GradientBoostingRegressor (m_p_, n_p_)
2 clf.fit (loss = 'squared_error', learn_r = 0.1, n_est = 100, subsample = 1.0, c = 'friedman_mse', m_s_s = 2, m_s_l = 1, max_depth = 3, alpha = 0.9, valid_frac = 0.1)
**Output:** Gradient Boosting Prediction model

In the above algorithm n_est represents n_estimators, c represents criterion, m_s_s represents min_sample_split, m_s_l represents min_samples_leaf, learn_r represents learning_rate, valid_frac represents the validation_fraction.

**AdaBoost model**: An AdaBoost regressor is a meta-estimator. It starts by fixing a prediction on the given dataset, after that fixes extra copy of the predictor to the coupled dataset (Agyemang et al., 2022). The instance weights are modified depends on the error of present regression. In essense, the successive predictors concentrate on hard instances. AdaBoosting regression technique is presented in Algorithm 4. The highest amount of estimates used till boosting is stopped is set as 50. The weight put into every predictor at every boosting epoch is called as learning rate. The increase in learning rate, improves the benefaction of every predictor. The learning rate is set as 1. After every boosting epoch, the weights are getting changed by loss function. The linear loss function is used.

**Algorithm 4:** AdaBoost model.

**Result:** Prediction of delamination size
**Input:** Sensor features with ground truth $\left( {{m_p},{n_p}} \right)_{p = 1}^x$
**1** clf = AdaBoostRegressor (m_p_, n_p_)
2 clf.fit ( n_estimators = 50, learning_rate = 1.0, loss = 'linear')
**Output:** AdaBoost Prediction model

In the above algorithm n_est represents n_estimators, learn_r represents learning_rate.

**Decision tree model**: Decision tree is a non-criterion supervised learning technique. The main is to produce a technique that regress the estimate of a desired variable through studying effortless decision rules worked out from the data features (Wang et al., 2019). The trees are known as piecewise constant imprecision. The decision trees study from data to imprecise a sine curve with group of if-then-else decision rules. The decision tree regression technique is presented in Algorithm 5. The method to calculate the standard of a split is known as criterion. Squared error criterion is used. The amount of samples needed to divide an internal node is set as 2 and amount of samples needed at leaf node is set as 1.

**Algorithm 5:** Decision tree model.

**Result:** Prediction of delamination size
**Input:** Sensor features with ground truth $\left( {{m_p},{n_p}} \right)_{p = 1}^x$
**1** clf = DecisionTreeRegressor (m_p_, n_p_)
2 clf.fit (criterion = 'squared_error', splitter = 'best', min_samples_split = 2, min_samples_leaf = 1)
**Output:** Decision Tree Prediction model

In the above algorithm c represents criterion, m_s_s represents min_sample_split, m_s_l represents min_samples_leaf.

**Ensemble model**: The ensemble technique takes on several base prediction techniques, whose regression accuracy is best than any other learning model. It is contrast from the ensemble technique in statistical devices, which is normally limitless. This ensemble-based ML technique increases the pliable structure of alternate technique who is finite (Kang & Cha, 2018).

This work used the stacking ensemble. It is an ambiguous loss-based ML framework. Stack ensemble comprises in stacking the output of separate regressor and utilize a predictor to calculate the end prediction. Stack ensemble permits to utilize the robustness of every separate predictor by utilizing their result as input to end predictor. The base regressor used for ensemble technique in this work is SVM, extra tree, gradient boosting, Adaboost and decision tree. Consequently, the base regressor techniques are implemented separately is presented in Algorithm 6.

**Algorithm 6:** Stacking ensemble model.

**Result:** Prediction of delamination size.
**Input:** Sensor features with ground truth $\left( {{m_p},{n_p}} \right)_{p = 1}^x$.
**Output:** Stacking ensemble E.
**1 Step 1: Develop base-level models CLF on E**
**2** Perform n-fold cross-validation on base level models
3 clf_1_ = svr (m_p_, n_p_)
4 clf_2_ = ExtraTreeRegressor (m_p_, n_p_)
5 clf_3_ = GradientBoostingRegressor (m_p_, n_p_)
6 clf_4_ = AdaBoostRegressor (m_p_, n_p_)
7 clf_5_ = DecisionTreeRegressor (m_p_, n_p_)
8 **Step 2: Construct the level-on data M**
9 $M = \left\{ {{{\hat m}_p},{n_p}} \right\}_{p = 1}^x,\; where$
10 ${\hat m_p}$ = {clf_1_(m_p_), clf_2_(m_p_), clf_3_(m_p_), clf_4_(m_p_), clf_5_(m_p_)}
11 Return E comprising of CLF models

## Results

To prove the efficiency of the proposed work, raw sensor data collected from one composite coupon is considered. The database was constructed with needed features for predicting delamination are ToF, cycle, frequency, PSD, and ground truth, *i.e*., delamination size. At final, the data set has 150,949 data points. From this, 75% data points are used to train the model and remaining 25% data points are used for testing. First, SVM regression technique was implemented with RBF kernel, but the prediction results were not preferable. Hence, further regression techniques like extra tree, gradient boost, AdaBoost decision tree were used to predict the delamination size. Finally, stack ensemble technique was used to combine the above said regression techniques.

MATLAB is used to process raw sensor data before building the data collection. Six piezoelectric actuators and six piezoelectric transducer (PZT) sensors are included in the composite coupon to collect raw data. Python scikit learn runs machine learning algorithms on an i5 processor, 8 GB of RAM, and Windows 10.

**Model estimation:**

To calculate the efficiency of machine learning model used for delamination prediction is estimated using root mean square error (RMSE) and coefficient of determination (R^2^). The formulas are as follows:

$RMSE = \sqrt {\displaystyle{1 \over x}\mathop \sum \limits_{i = 1}^x {{\left( {{p_i} - \; {{\hat p}_i}} \right)}^2}} \; \; \; \; \; \; \; \; \; \; \; \; \; \; \; \; \; \; \; \; \; \; \; \; \; \; \; \; \; \; \; \; \; \; \; \; \left( 2 \right)$

${R^2} = 1 - \displaystyle{{\mathop \sum \nolimits_{i = 1}^x {{\left( {{p_i} - {{\hat p}_i}} \right)}^2}} \over {\mathop \sum \nolimits_{i = 1}^x {{\left( {{p_i} - {{\bar p}_i}} \right)}^2}}}\; \; \; \; \; \; \; \; \; \; \; \; \; \; \; \; \; \; \; \; \; \; \; \; \; \; \; \; \; \; \; \; \; \; \; \; \; \; \; \left( 3 \right)$

where RMSE is absolute estimate to fit, the less RMSE is best estimate to fit and R^2^ is a relative measure to fit, it varies from 0 to 1, the high R^2^ specify a better model.

**Figure 7:**
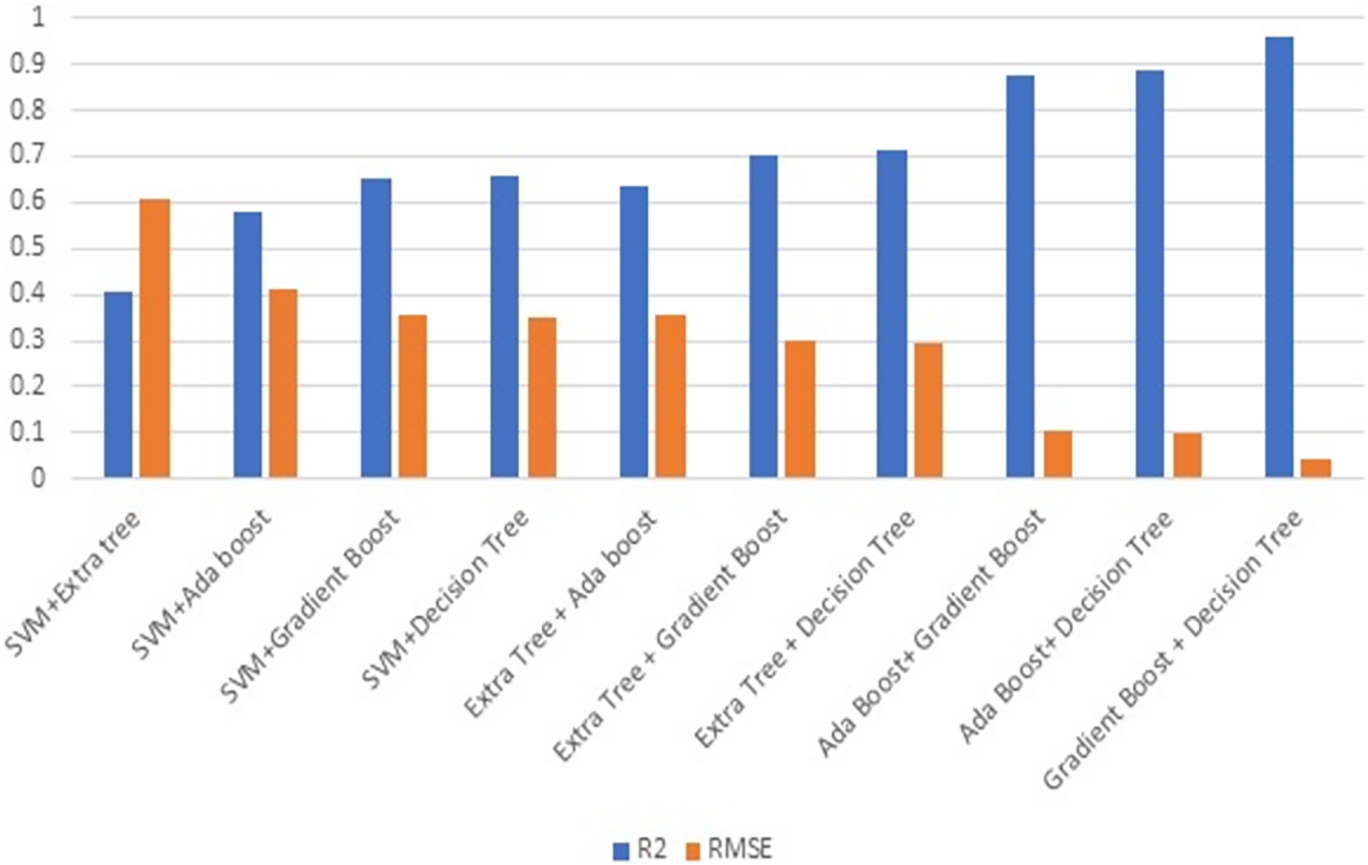
Ensembling using two methods.

Figure 7 illustrates the R2 and RMSE values when combining two ML techniques. Ensembling is the combination of one or more approaches that enhance the outcome of the SHM procedure. Ensembles are very good at preventing overfitting, improving generalization, and handling noisy or inconsistent data. They provide a robust solution to a wide range of datasets, as different models may thrive in different areas of feature space. Furthermore, ensemble approaches are less susceptible to hyperparameter tuning and outliers, making them more durable and adaptive to a variety of real-world circumstances. Overall, the diversity and aggregation of numerous models inside an ensemble framework result in more robust, accurate, and reliable predictions in machine learning applications. This stage involves evaluating the efficiency of combining two strategies. Combining gradient boost with decision tree surpasses all other models in terms of maximising R2 and decreasing RMSE.

**Figure 8:**
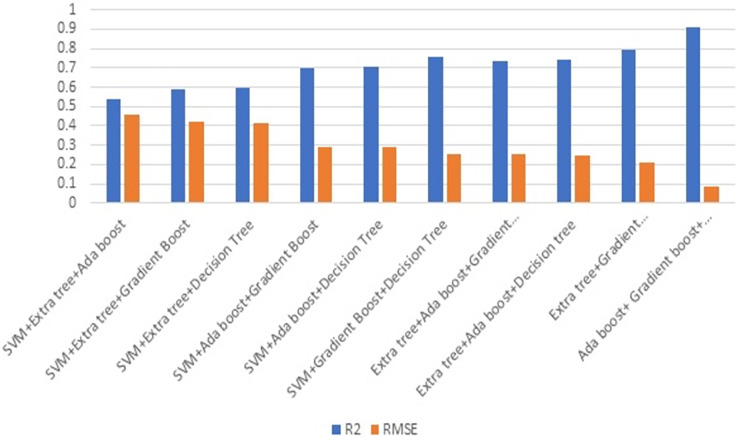
Ensembling using three methods.

Figure 8 illustrates the effectiveness of combining three strategies. The comparison of Figs. 7 and 8 illustrates the performance, which demonstrates that gradient boost combined with other approaches delivers superior results in comparison to other approaches. Similarly, the combination of three ML methods does not outperform the combination of two ML methods. This demonstrates that the combination of three ML approaches does not always yield positive results. This combination of ML techniques yields results dependent on the characteristics of the dataset.

**Figure 9:**
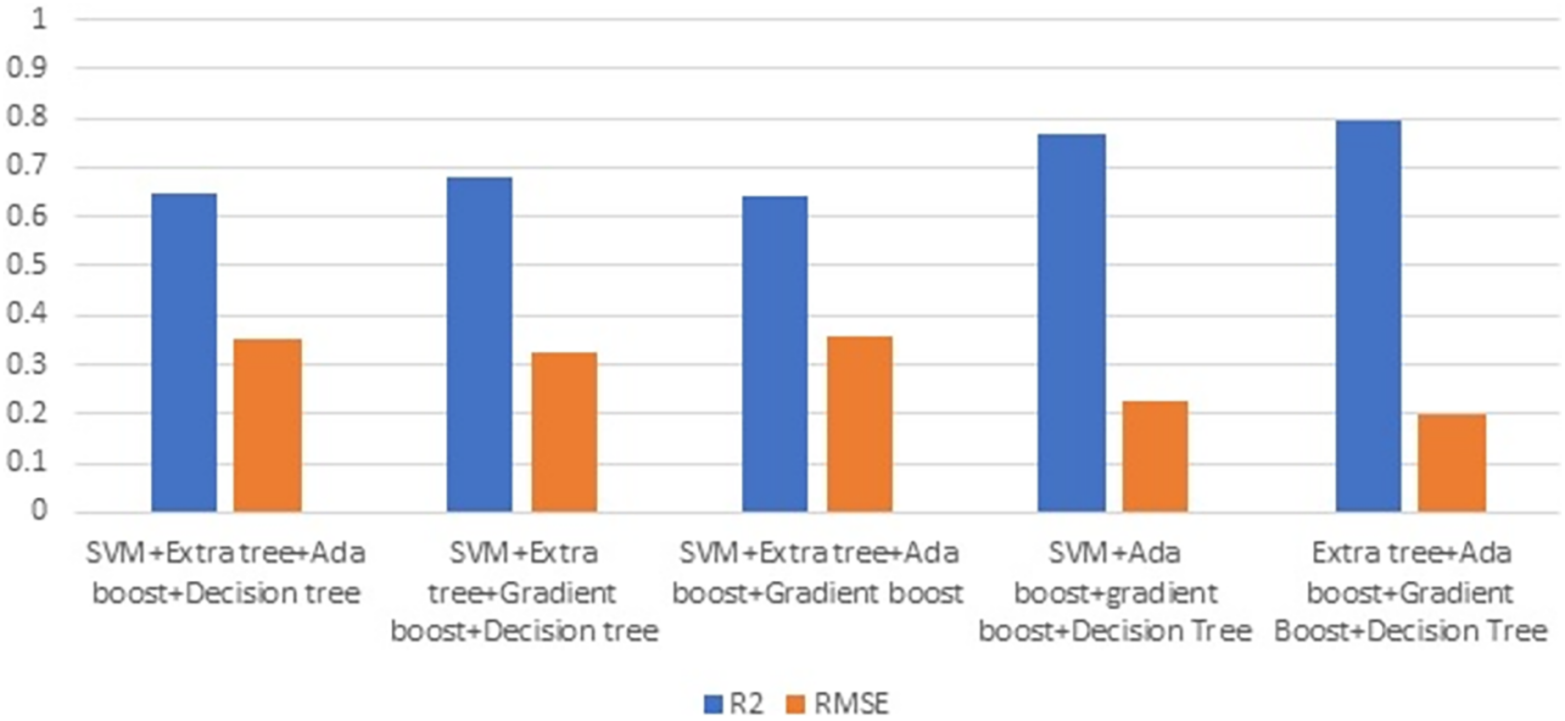
Ensemble using four methods.

Figure 9 depicts the result of combining four methods. Combining three ML techniques and four ML techniques. Observing Figs. 8 and 9 demonstrates conclusively that integrating multiple ML algorithms does not produce optimal results for all datasets. Before utilising ensembling techniques, thoroughly examine the test data and then use the appropriate combination of ML algorithms.

After analysing each and every test data, combination of five ML approaches forms an ensembling approach. The combination ML methods (SVM, extra tree, AdaBoost, gradient boost and decision tree) outperforms the best result compared to individual ML methods as well as combination two, three and four ML methods. The evidence is provided in the Table 1.

**Table 1:** Model evaluation.

Model	R^2^	RMSE
SVM	0.352	0.662
Extra tree	0.462	0.548
AdaBoost	0.806	0.159
Gradient boost	0.948	0.050
Decision tree	0.967	0.040
Ensemble	0.975	0.023

Table 1 represents the evaluation result of each separate model and stacking (ensemble) model. The evaluation result shows that ensemble model outperforms all the single model with lowest RMSE and highest R^2^ value. Table 2 represents the evaluation result of each model and stacking ensemble model for a new composite coupon which was not trained yet. The new composite coupon is made up of different materials and tries to check the performance of ensembling techniques.

**Table 2:** Model evaluation for new coupon.

Model	R^2^	RMSE
SVM	0.319	0.694
Extra tree	0.423	0.586
AdaBoost	0.785	0.181
Gradient boost	0.907	0.087
Decision tree	0.913	0.078
Ensemble	0.928	0.053

Several cause for error in the accuracy of prediction are delamination area calculation (ground truth) in MATLAB. Sensed signal orientation, external noise affected the sensed signal and less amount of data. Also, there are some technical difficulties for constructing the data set which may cause some error in delamination size prediction.

**Figure 10:**
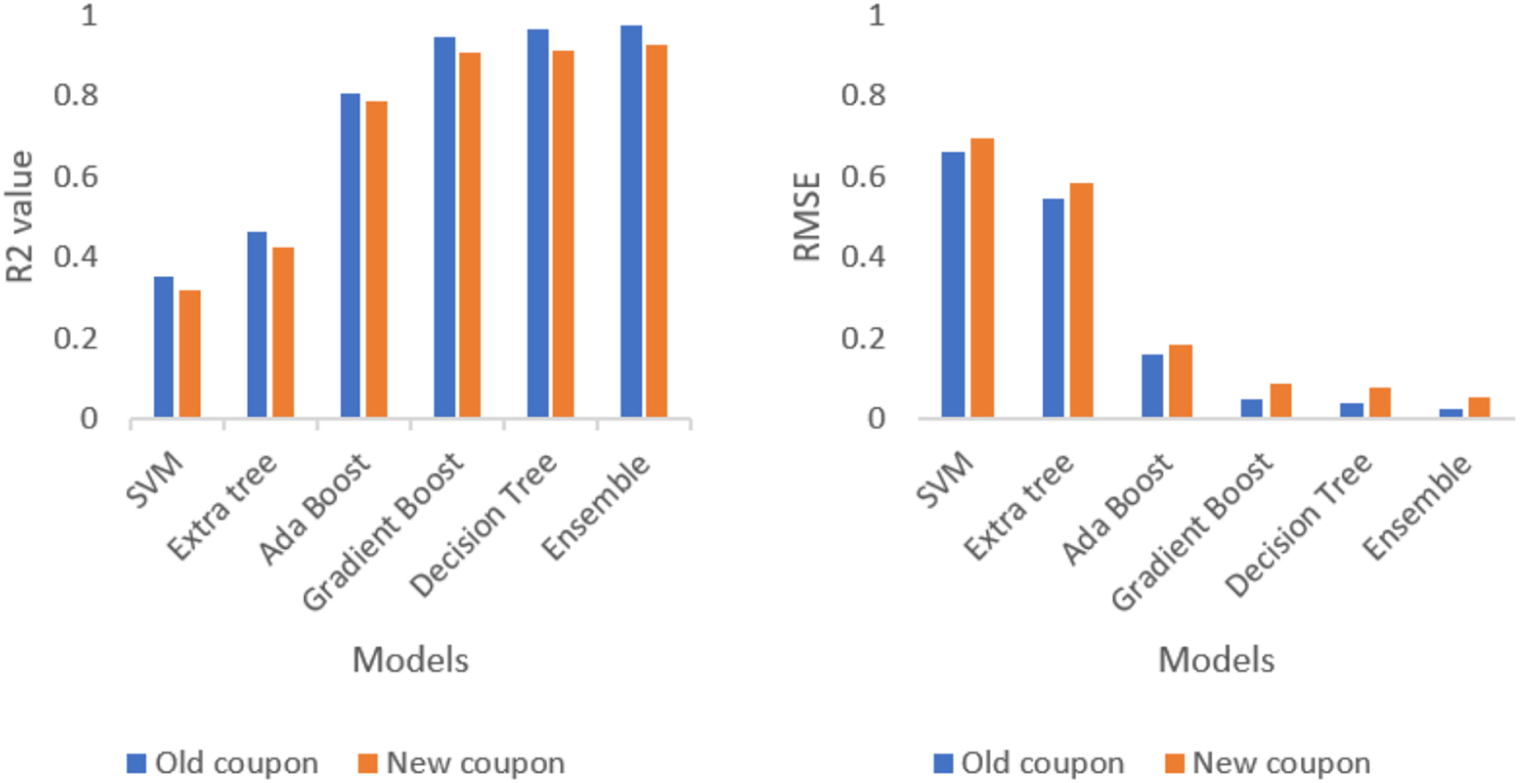
Comparison of R^2^ value and RMSE value for old and new coupon.

Ensembling techniques are tested against the new coupon and analyse the performance metrics of R^2^ and RMSE. The comparison of new coupon and old coupon are displayed in the Fig. 10. Even though the prediction accuracy is less than old coupon, the ensemble model outperforms all the single model with lowest RMSE and highest R^2^ value.

## Discussion

The experimental assessment shows an efficient technique for delamination prediction using machine learning model. In this research work, ensemble technique produces better accuracy with error rate, because ensemble technique has the strength of each regression technique and acted better than each technique.

The SVM, in particular, wraps the perseverance of the variables for a given usefulness of the method, kernel variables, and kernel possibility. The SVM technique assures the difficulty of overfitting from variable enhancement to procedure choice. Nonetheless, kernel approaches will be entirely diplomatic in terms of overfitting the technique determination criteria. In a decision tree, it will be difficult to evaluate all possible attribute combinations in order to find unseen data with deprecatory failure. The decision tree focuses on discovering errors by distinguishing between success and error data. Extra trees are typically powerful for discrepancy. Anyway, due of its proclivity for overfitting, it is prone to sampling errors. When the testing data set differs significantly from the training data set, the extra tree cannot be fitted. Overfitting is possible with boosting approaches (gradient and AdaBoost), and the maximum number of regression trees is not allowed for one.

Each regression technique has its own advantage and disadvantage when these features are interrelated. Accordingly, the stacking ensemble technique, take in from each regression technique’s advantages to balance their disadvantages, accomplishing correctly in together or more than the best individual technique with reference to improving the prediction accuracy. The main strength of the stacking ensemble model is, considered each separate regression technique and taken their advantage and produced better accuracy for given data set.

## Conclusions

The primary outcome of this research is to focusses on finding the suitable ML algorithm to predict the delamination size in the structure of the aircraft. The work represented in this article focuses on construct a damage assessment technique for structural health monitoring of aircraft. In this article, the damage assessment mainly aims in designate the increase of delamination in composite materials. This work shown a innovative approach to identify the damaged area through delamination size prediction with machine learning model. Five machine learning techniques with stacking ensemble approach were used to identify the size of delamination in a composite coupon. Analysed the results produced by SVM, extra tree, AdaBoost, gradient boost, decision tree and stacking ensemble technique, the stacking ensemble method outperformed all the technique with 0.975 R^2^ and 0.023 RMSE for old coupon and 0.928 R^2^ and 0.053 RMSE for new coupon. It shown the increase in R^2^ and decrease in root mean square error (RMSE).

The features frequency, cycle, ToF and PSD alone considered in this article. Adding more features will increase the performance. Other than delamination, skin stiffener, matrix cracking, stain patterns, fiber failure also need to be considered while monitoring the structural heath of aircraft. These things will be concentrated in future work.

## Supplemental Information

10.7717/peerj-cs.1955/supp-1 Supplemental Information 1 Code.

10.7717/peerj-cs.1955/supp-2 Supplemental Information 2 Dataset.

## Additional Information and Declarations

### Data Availability

The following information was supplied regarding data availability: The Carbon Fiber-Reinforced Polymer (CFRP) Composites is available at NASA: https://www.nasa.gov/intelligent-systems-division/discovery-and-systems-health/pcoe/pcoe-data-set-repository. The processed final data set and the code are available in the Supplemental Files.
